# NSD1 inactivation defines an immune cold, DNA hypomethylated subtype in squamous cell carcinoma

**DOI:** 10.1038/s41598-017-17298-x

**Published:** 2017-12-06

**Authors:** Kevin Brennan, June Ho Shin, Joshua K. Tay, Marcos Prunello, Andrew J. Gentles, John B. Sunwoo, Olivier Gevaert

**Affiliations:** 1 Department of Medicine, Stanford Center for Biomedical Informatics Research, Stanford University, Stanford, USA; 2 Department of Otolaryngology – Head and Neck Surgery, Stanford University School of Medicine, Stanford, USA; 3 Department of Statistics, College of Pharmaceutical and Biochemical Sciences, National University of Rosario, Rosario, Argentina; 4 Department of Otolaryngology – Head and Neck Surgery, National University Health System, Singapore, Singapore

## Abstract

Chromatin modifying enzymes are frequently mutated in cancer, resulting in widespread epigenetic deregulation. Recent reports indicate that inactivating mutations in the histone methyltransferase NSD1 define an intrinsic subtype of head and neck squamous cell carcinoma (HNSC) that features pronounced DNA hypomethylation. Here, we describe a similar hypomethylated subtype of lung squamous cell carcinoma (LUSC) that is enriched for both inactivating mutations and deletions in *NSD1*. The ‘NSD1 subtypes’ of HNSC and LUSC are highly correlated at the DNA methylation and gene expression levels, featuring ectopic expression of developmental transcription factors and genes that are also hypomethylated in Sotos syndrome, a congenital disorder caused by germline *NSD1* mutations. Further, the NSD1 subtype of HNSC displays an ‘immune cold’ phenotype characterized by low infiltration of tumor-associated leukocytes, particularly macrophages and CD8^+^ T cells, as well as low expression of genes encoding the immunotherapy target PD-1 immune checkpoint receptor and its ligands. Using an *in vivo* model, we demonstrate that NSD1 inactivation results in reduced T cell infiltration into the tumor microenvironment, implicating NSD1 as a tumor cell-intrinsic driver of an immune cold phenotype. NSD1 inactivation therefore causes epigenetic deregulation across cancer sites, and has implications for immunotherapy.

## Introduction

Nuclear receptor binding SET domain protein 1 (*NSD1*) is frequently mutated in head and neck squamous cell carcinoma (HNSC)^1,2^, the sixth most common cancer by incidence^3^, and a leading cause of cancer-related death^4^. NSD1 is also genetically or epigenetically deregulated (either inactivated or overexpressed) in several other cancer types^1,2,5–12^.

NSD1 is best known as the causative gene for the congenital overgrowth disorder Sotos syndrome, which is associated with mildly increased cancer incidence^13–15^. NSD1 is therefore among several epigenetic modifying enzymes (such as NSD2, DNMT3a, SETD2, EZH2) that represent causative genes for developmental growth disorders that are also frequently mutated in cancer^16^.

*NSD1* is a SET-domain containing histone methylatransferase, which catalyzes methylation of histone 3 at lysine 36 (H3K36). Current evidence suggests that NSD1 catalyzes H3K36 dimethylation (H3K36me2)^17–19^, though the precise epigenetic function of NSD1 (i.e. the H3K36 methylation states it catalyzes, its target genes and genomic loci, and the functional consequence of these marks) remains largely unknown.

Choufani *et al*. reported that germline *NSD1* mutations are associated with widespread perturbation (primarily loss) of DNA methylation^20^, i.e., methylation of cytosine to form 5-methylcytosine at CpG dinucleotides. NSD1 is not thought to methylate DNA; therefore H3K36me (or other histone marks) catalyzed by NSD1 apparently regulate DNA methylation.

Inactivating mutations of *NSD1* also deregulate DNA methylation in HNSC, as we and others have described a HNSC subtype characterized by widespread DNA hypomethylation, that is strongly enriched for NSD1 mutations^2,19,21^. We recently identified this ‘NSD1 subtype’ as one of five HNSC DNA methylation subtypes, using data from 528 HNSC patients from The Cancer Genome Atlas (TCGA) study^2,22^. Papillon-Cavanagh *et al*. recently reported that a HNSC subtype featuring NSD1 mutations is defined by impairment of dimethylation (H3K36me2) and that NSD1 inactivation represents one of two mechanisms causing H3K36me2 impairment, the other being H3 K36M mutations^19^. These findings reveal NSD1 inactivation as one mechanism underlying deregulation of DNA methylation, a major cause of abnormal gene expression in virtually all cancers^16^.

Analysis of the gene expression profiles of these subtypes indicated striking inter-subtype differences in the profiles of both overall and cell type-specific tumor associated leukocytes (TALs). Tumors can exploit mechanisms of immune regulation to suppress infiltration of immune cells into the tumor microenvironment, thus avoiding anti-tumor immunity. There is a growing interest in identifying these mechanisms, which may be targeted using immunotherapies to restore innate anti-tumor immunity. Immunotherapies provide particular promise for metastatic HNSC; however they are only effective in a subset of individuals, and are associated with autoimmune side effects, therefore there is clinical need for biomarkers to identify patients that may be particularly sensitive. Current evidence indicates that ‘immune hot’ tumors, particularly those with greater numbers of infiltrating PD-1^+^ or? CD8^+^ T cells, are more responsive to immunotherapy^23^, indicating that susceptibility to some immunotherapy approaches may vary between the HNSC subtypes.

Here, we follow up upon our subtyping analysis to describe the NSD1 subtype and report our identification of an epigenetically and transcriptionally similar NSD1 subtype occurring in lung squamous cell carcinoma (LUSC). We further investigated the immune profile of the HNSC NSD1 subtype and found that it represents an ‘immune cold’ subtype, with the lowest levels of overall and cell type-specific immune infiltrating lymphocytes among the five different HNSC tumor subtypes. We demonstrate that NSD1 inactivation induces immune cell exclusion from the tumor microenvironment using an *in vivo* mouse model of tumor immune infiltration, recapitulating the immune cold phenotype observed in the analysis of the TCGA data. These results may have important implications as a biomarker for the future selection of immune therapy-responsive patients.

## Results

### Association of NSD1 mutations and deletions with a DNA hypomethylated HNSC subtype

We recently described a HNSC subtype featuring widespread DNA hypomethylation co-occurring with NSD1 mutations using MethylMix^21,22^. Of 2,602 genes found to be abnormally methylated in HNSC relative to normal tissue overall^22^, 1127 were significantly hypomethylated, and 102 hypermethylated, in the *NSD1* subtype relative to other HNSC subtypes combined (Supplementary Tables 1 & 2). Fifty-seven percent (24/42) of patients within this HNSC subtype had *NSD1* mutations, compared with 2–8% patients within the other subtypes. This subtype included all five patients with ‘high-level’ somatic deletions called by GISTIC 2.0^24^, as well as enrichment of ‘low-level’ deletions. NSD1 deletions were significantly enriched among patients with NSD1 point mutations, as 21/33 (64%) of patients with NSD1 mutations had deletions, compared with 99/269 (0.34) of patients without mutations. However, mutations and deletions were each independently associated with both *NSD1* RNA expression (Supplementary Figure 1a) and mean DNA methylation across all abnormally methylated genes (Supplementary Figure 1b). Lowest NSD1 expression and mean methylation occurred in patients with high-level likely focal deletions but without mutations, and in patients with both NSD1 mutations and deletions, suggesting that tumors undergo positive selection for loss of both alleles, resulting in extreme hypomethylation. Moreover, patients with low-level deletions (CNV = −1) had significantly lower mean DNA methylation than patients with normal copy number (CNV = 0), both in patients with and without NSD1 mutations, indicating that NSD1 deletions impair DNA methylation independent of mutations.

### Identification of a hypomethylated, NSD1 inactivated subtype of lung squamous cell carcinoma

We investigated the possibility that NSD1 mutations affect DNA methylation in other cancers, focusing on cancers for which there were at least ten patients with *NSD1* mutations and accompanying DNA methylation data within TCGA data. These included LUSC, uterine corpus endometrial carcinoma (UCEC), and breast carcinoma (BRCA). LUSC was the only of these cancers in which NSD1 mutations were significantly associated with DNA hypomethylation (p = 0.001) (Supplementary Figure 2).

To investigate whether NSD1 inactivation occurred within a hypomethylated subtype of LUSC, we identified LUSC subtypes using the same method that was previously used to identify the HNSC subtypes^25^: We applied MethylMix to 503 LUSC patients to identify abnormally methylated genes (n = 3,025 genes identified), and then applied consensus clustering to the DNA methylation states of these genes (An output of MethylMix) to identify clusters of patients with homogenous DNA methylation profiles. This method revealed six clusters, or putative subtypes.

**Figure 1:**
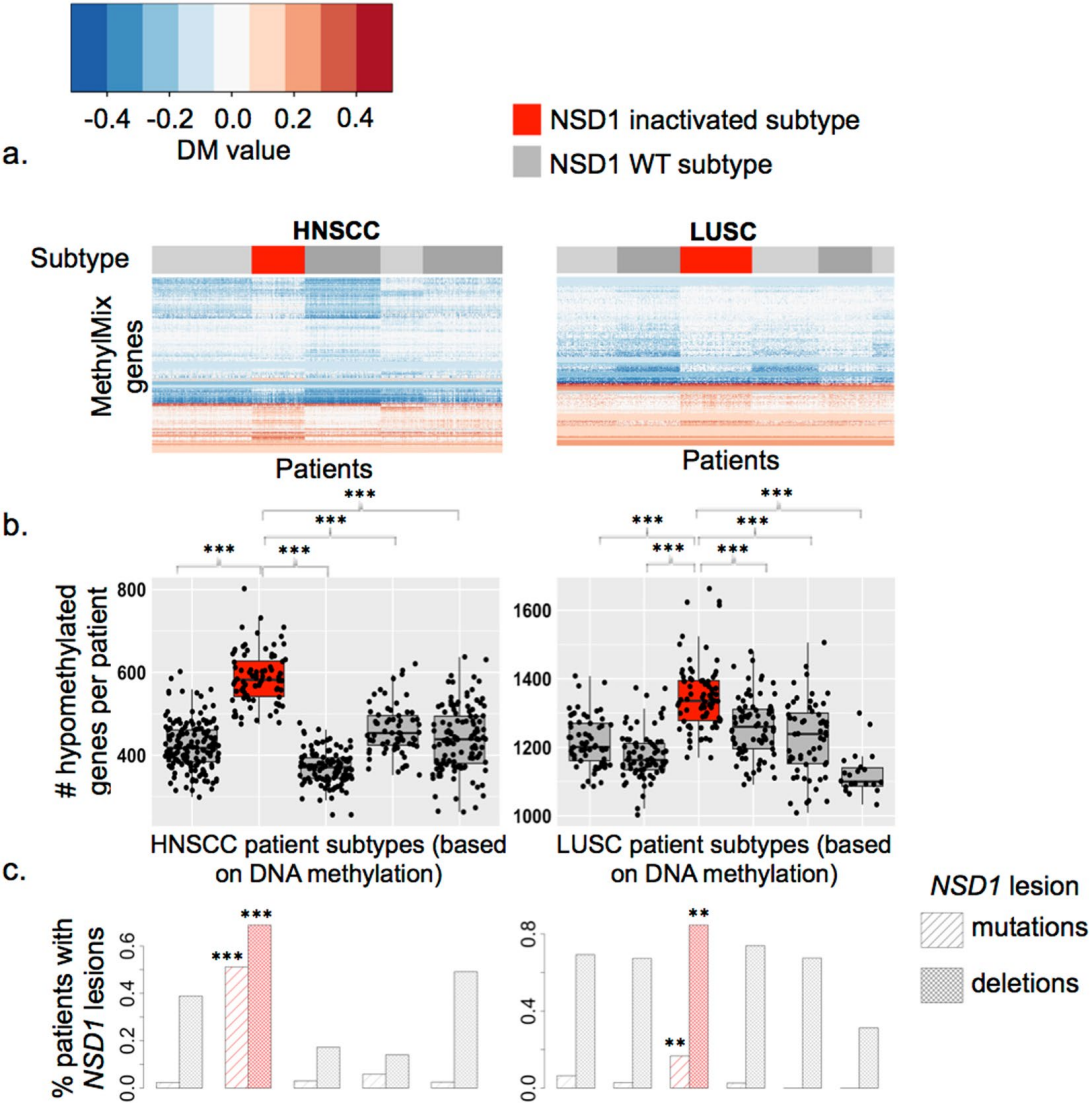
Identification of NSD1 inactivated subtypes of squamous cell carcinomas featuring epigenetic de-repression of developmental oncogenes: (**a**) Heatmaps of differential methylation (DM) values illustrate five subtypes of head and neck squamous cell carcinoma (HNSC, n = 528 patients) and six subtypes of lung squamous cell carcinoma (LUSC, n = 502 patients) within TCGA studies, identified by consensus clustering of patients according to their profiles of abnormally methylated genes, subsequent to identification of these abnormally methylated genes by applying MethylMix to integrate DNA methylation and gene expression data. DM values represent the beta value difference in methylation between tumor and normal adjacent tissue for hypomethylated (<0) or hypermethylated (>0) methylation states for each gene, calculated by MethylMix^71^. Red bars demarcate hypomethylated NSD1 subtypes, while light and dark grey bars demarcate other subtypes. (**b**) The average number of genes hypomethylated per patient (in tumor relative to normal tissue) was significantly higher in NSD1 subtypes (red) than each other subtype (grey) in both HNSC and LUSC. (**c**) Percentages of patients within each subtype that have NSD1 mutations (Striped bars) and NSD1 deletions (Solid bars) in HNSC and LUSC. Asterisks indicate the significance of enrichment of NSD1 mutations or deletions within the NSD1 subtype (red) compared with patients in all other subtypes (Pearson’s chi-squared test).

One of these subtypes had a significantly elevated number of hypomethylated genes (Fig. 1). This subtype included six of ten LUSC patients with NSD1 mutations, representing 17% of patients in this subtype (p = 0.005). This subtype was also enriched for NSD1 deletions, as 88/104 (84%) of patients within this subtype had deletions compared with 31–74% patients within other subtypes (p = 0.001). NSD1 RNA expression and DNA methylation displayed the same inverse trend with mutations and deletions, as seen in HNSC (Supplementary Figure 1).

**Figure 2:**
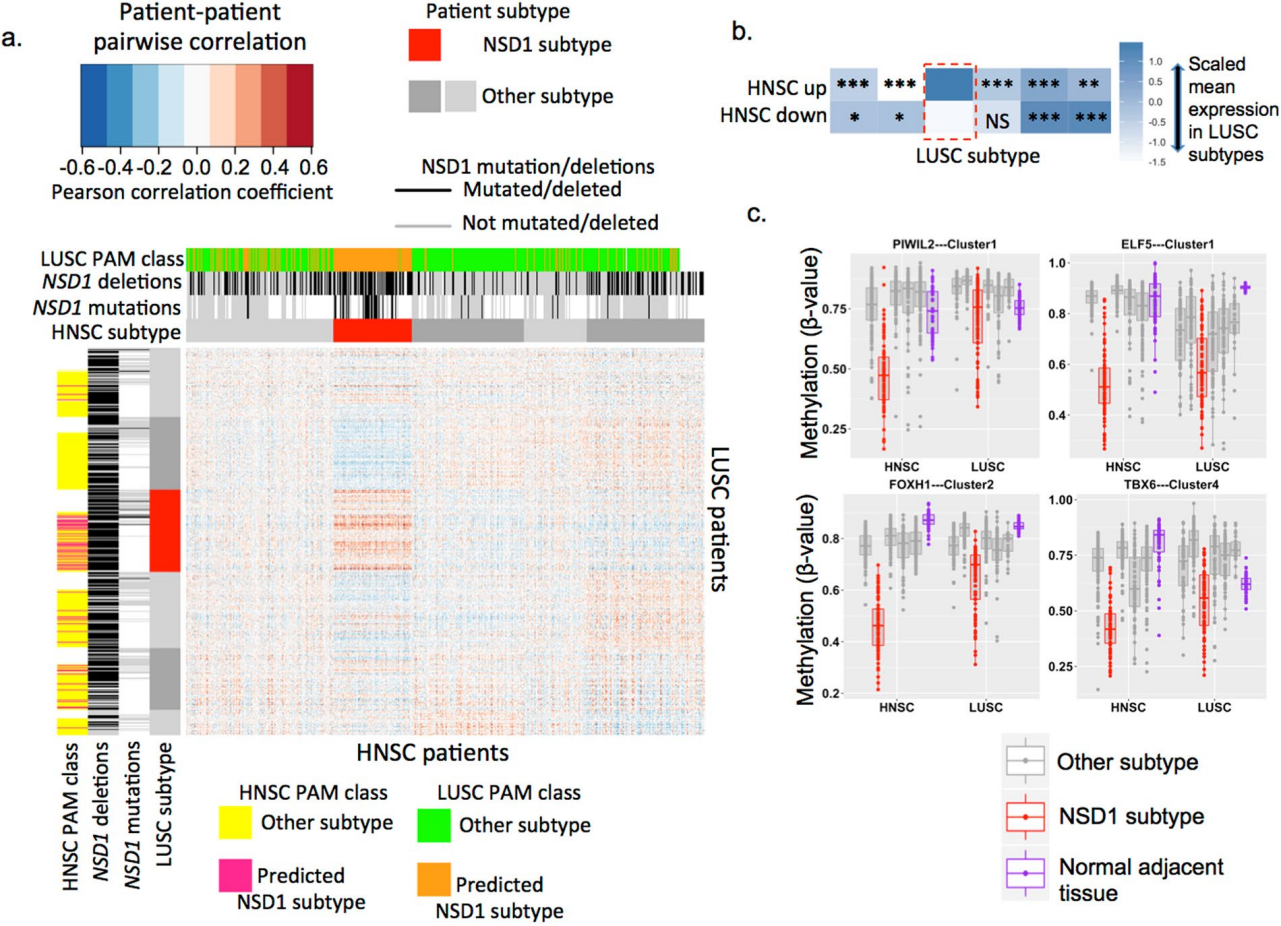
Concordant DNA methylation and gene profiles between HNSC and LUSC subtypes: (**a**) Heatmap of a correlation matrix illustrating pair-wise Pearson correlations of DNA methylation profiles between 528 HNSC patients (columns) and 503 LUSC patients (rows). Correlation coefficients indicate the correlation of each patient pair across 621 CpG sites, representing all CpG sites that were available for all patients (Measured on both Illumina 27 k and 450 k arrays), and which were abnormally methylated (hypermethylated in hypomethylated) in all or a subset of HNSCs. Patients are ordered according to DNA methylation subtypes (no clustering was performed), with sidebars indicate HNSC and LUSC subtypes (NSD1 subtypes illustrated in red, other subtypes grey). NSD1 mutation and deletion sidebars indicate patients with NSD1 mutations or deletions (black), absence of NSD1 mutations or deletions (grey), or missing data (white). The ‘NSD1 PAM class’ sidebar indicates predictions of PAM models for belonging to the NSD1 subtype, which were trained on one cancer type and used to classify patients of the other cancer type as either ‘NSD1 subtype’, or ‘Other subtype’ (e.g., ‘HNSC PAM class’ refers to predictions made by a model trained on HNSC subtypes and applied to predict subtypes of LUSC patients). (**b**) Scaled mean RNA expression in LUSC DNA methylation subtypes of genes that were upregulated (HNSC up) and downregulated (HNSC down) in the HNSC NSD1 subtype. Asterisks indicate the significance of differential mean expression between the NSD1 LUSC subtype (Red box) and each other subtype (Wilcoxon rank sum test): NS Not significant, *P < 0.05, **P < 0.01, ***P < 0.001. (**c**) DNA methylation of development-related transcription factor genes, in normal tumor-adjacent tissue (purple), and in tumor of patients within NSD1 subtypes (red) or other subtypes (grey), in HNSC and LUSC.

The DNA methylation profiles of the HNSC and LUSC NSD1 subtypes were strongly concordant, illustrated by a correlation matrix heatmap indicating pairwise correlations between each HNSC patients and LUSC patients (Fig. 2a). Previous investigations have identified concordance between HNSC and LUSC gene expression subtypes, using centroid predictor based approaches^2,26^. We used a similar method, PAM analysis^27^, to classify those LUSC patients that are similar to the HNSC NSD1 subtype, and HNSC patients that are similar to the LUSC NSD1 subtype, based on their DNA methylation profiles. We first trained PAM models to classify the NSD1 subtypes in HNSC and LUSC separately. For each cancer site, we used PAM in combination with 10-fold cross validation to determine the ability of DNA methylation data to classify NSD1 subtype patients. For each fold of cross validation, the PAM model was trained on 90% of patients (Training set) and assigned class probability (Probability of belonging to the NSD1 subtype) to each of the remaining 10% of patients (Test set). We used the Area under the ROC curve (AUC) to evaluate the performance of the models in classifying NSD1 subtype patients, indicating the mean classification error rate across the ten folds of cross validation. These PAM models for HNSC and LUSC could classify NSD1 subtype patients with areas under the receiver-operating curve (AUC) of 0.997 (95% CI: 0.991–1), and 0.86 (95% CI: 0.81–0.90), respectively. The AUC for the HNSC PAM model remained high (0.96 (95% CI: 0.94–0.99)) when the number of CpG sites used for class prediction was reduced to just five, indicating that it would be possible to identify the HNSC NSD1 subtype using a minimal CpG panel biomarker. A largely consistent set of CpG sites was selected by the model to predict the NSD1 subtype repeatedly across each fold of cross-validation, with nine CpGs used overall. These ‘highly predictive’ CpGs were all highly methylated in normal adjacent tissue and specifically hypomethylated in tumors of the NSD1 subtype, and are provided in Supplementary Table 3 (lines 185–188).

We validated the HNSC PAM model by applying it to an independent set of 44 primary HNSCs, for which methylation, RNA expression and copy number data was available (GSE33232)^28^. Six (14%) of these HNSCs that were classified as the NSD1 subtype. These predicted NSD1 subtype patients had significantly lower *NSD1* RNA expression compared with those not predicted as belonging to the NSD1 subtype (p = 0.014, Supplementary Figure 3). Interestingly, *NSD1* RNA expression was negatively correlated with methylation of genes that were hypermethylated in the HNSC subtype, as well as positively correlated with genes that were hypomethylated, confirming that NSD1 inactivation causes DNA hypermethylation as well as hypomethylation. Both patients with *NSD1* deletions were within the group predicted as the NSD1 subtype. This indicated that the HNSC NSD1 subtype PAM model could classify NSD1 subtype patients in external data sets.

We next applied the HNSC PAM model to LUSC patients, and found that 58/365 (16%) of patients were assigned to the HNSC NSD1 subtype class, of which 35 (60%) were within the LUSC NSD1 subtype, representing a strong enrichment (p = 5.6e-15) (Fig. 2a). Conversely, when we applied the LUSC PAM model to HNSC, 165/527 (31%) of patients were assigned to the LUSC NSD1 subtype class, of which 79 (48%) were within the HNSC NSD1 subtype (p < 2.2e-16) (Fig. 2a). This confirmed the similarity of the HNSC and LUSC NSD1 subtypes at the DNA methylation level.

The HNSC and LUSC NSD1 subtypes were also concordant at the transcriptional level, as mean expression of genes upregulated and downregulated in the HNSC NSD1 subtype were upregulated and downregulated, respectively, in the LUSC NSD1 subtype, compared with each other subtype (Fig. 2b). The molecular similarity of the HNSC and LUSC NSD1 subtypes was primarily driven by DNA hypomethylation concordant with transcriptional upregulation, as 178/867 (20%) genes that were significantly overexpressed within the HNSC NSD1 subtype were also overexpressed within the LUSC NSD1 subtype (Supplementary Table 1), while 37/722 (5%) genes underexpressed with the HNSC NSD1 subtype were underexpressed within the LUSC NSD1 subtype (Supplementary Table 2).

When gene set enrichment analysis was performed for genes that were hypomethylated and overexpressed in both HNSC and LUSC, the most enriched gene set was represented genes that bear the activating histone mark H3K4me3 at their promoters in embryonic stem cells^29^, i.e. genes with an epigenetically active state in stem cells. Moreover, NSD1 subtypes featured hypomethylation and overexpression of transcription factors that are normally expressed specifically in germline tissues or during development, for example, *PIWIL2* ^30,31^, *ELF5* ^32^, *TBX6* ^33^ and *FOXH1* ^34^. These genes were highly methylated in adjacent normal tissues, but hypomethylated at functional gene regions, often promoter CpG islands (Supplementary Table 1), specifically within NSD1 subtypes.

We performed exploratory analyses to identify additional genes that are mutated and/or subject to copy number aberration within the NSD1 subtypes of HNSC or LUSC, in order to identify events that may cause hypomethylation in combination with NSD1 inactivation, or in NSD1 subtype patients that lack NSD1 lesion. We did not identify any such events (Data not shown).

### The cancer NSD1 DNA hypomethylation signature overlaps with the Sotos syndrome hypomethylation signature

Using a reported set of CpG sites that are abnormally methylated in Sotos syndrome^20^, we investigated the possibility that a shared set of genes is epigenetically deregulated by NSD1 in different diseases. Of 49 CpG probes hypermethylated in Sotos syndrome, none were hypermethylated in either HNSC or LUSC. However, of 7,038 probes hypomethylated in Sotos syndrome, 117 were hypomethylated in the HNSC NSD1 subtype, and 161 were hypomethylated in the LUSC NSD1 subtypes, with 54 hypomethylated probes within 31 unique genes overlapping between Sotos syndrome, HNSC and LUSC (p < 2.2e-16) (Supplementary Table 1).

To test the significance of overlap between the hypomethylated CpG signatures associated with NSD1 inactivation in cancer and Sotos syndrome, we calculated an index of overlap between the Sotos syndrome hypomethylated CpG signature and cancer hypomethylated CpGs in each patient. For each patient, we calculated the number of hypomethylated CpGs (hypomethylated in tumor relative to adjacent normal tissue) that overlapped with the Sotos syndrome hypomethylated CpG signature, and expressed this as a fraction of the overall number of hypomethylated CpGs. This ‘Sotos syndrome overlap index’ is therefore normalized for the overall number of hypomethylated CpG probes in each cancer. As a control, we generated a generated a ‘random overlap index’ by iteratively calculating the overlap with a random selection of 7,038 CpGs (the same length as the Sotos syndrome hypomethylated CpG signature) (See methods).

Median levels of the Sotos syndrome overlap index, but not the random overlap index, increased incrementally with and increasing number of inactivating NSD1 lesions (Mutations and deletions) in both HNSC and LUSC (Supplementary Figure 5). To formally test effect of NSD1 inactivation on the Sotos syndrome overlap index, we combined NSD1 mutations and deletions into a single ‘NSD1 lesion score’ (See methods for details) and tested for a linear association between this score and the Sotos syndrome overlap index. The Sotos syndrome overlap index increased with increasing number of inactivating NSD1 lesions in both HNSC and LUSC (Supplementary Figure 5a), and was higher in the NSD1 DNA methylation subtype compared with other subtypes in HNSC, though not in LUSC (Supplementary 6b). Overall, this analysis indicates similarity between the hypomethylation signatures associated with NSD1 inactivation in cancer and Sotos syndrome.

### NSD1 inactivation is associated with an immune cold phenotype in HNSC

**Figure 3:**
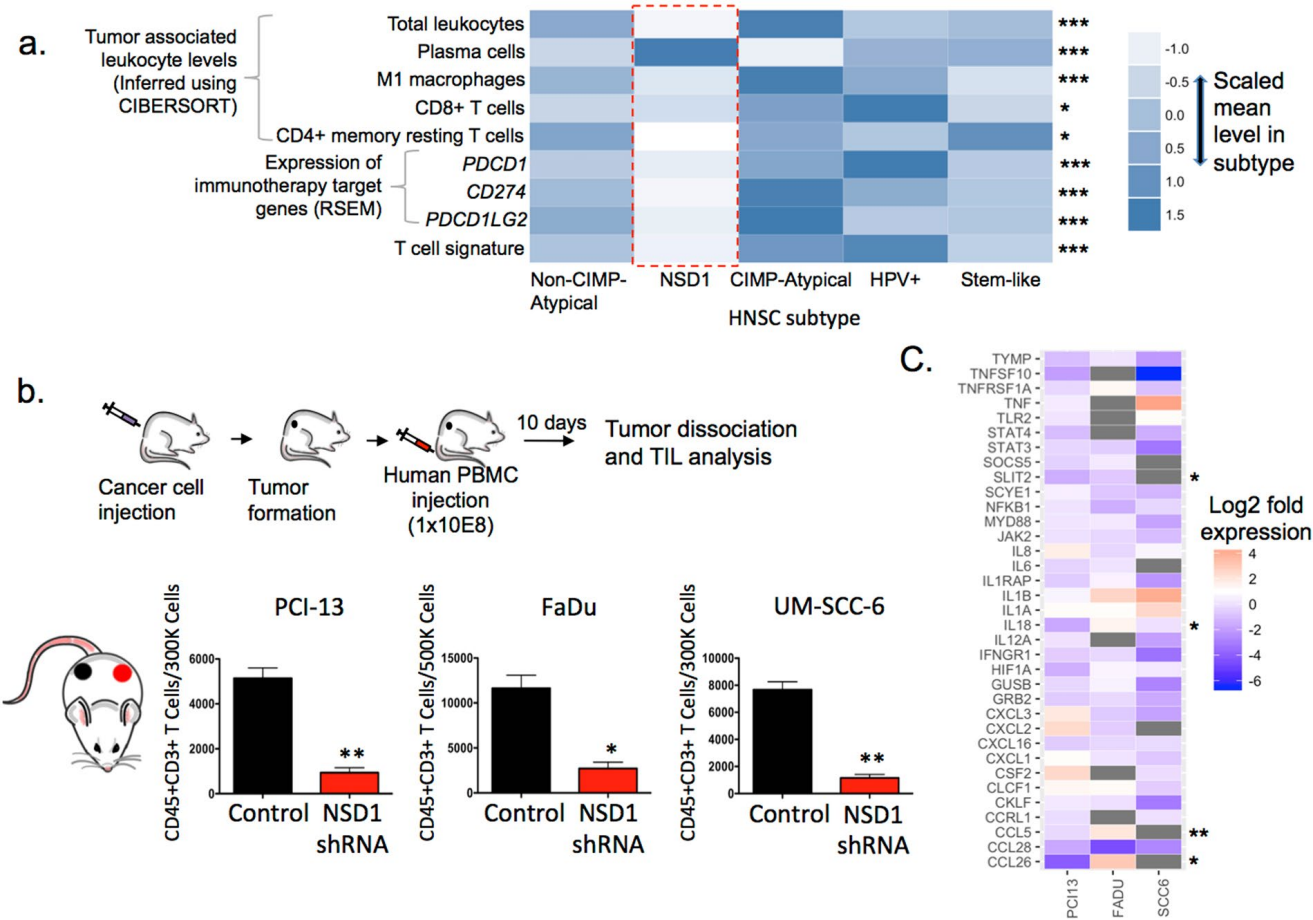
NSD1 inactivation is associated with immune cell exclusion from the tumor microenvironment in HNSC: (**a**) Compared with other HNSC subtypes, the NSD1 subtype (red box) displayed significantly lower mean signature levels of overall tumor associated leukocytes (TALs), and specific TAL types including M1 tumor associated macrophages (TAMs), CD8+ cytotoxic T cells, and CD4+ memory T cells (All inferred using CIBERSORT^36^). The NSD1 subtype had the low mean RNA expression of immunotherapy-relevant genes, including CD274 (PD-L1), PDCD1 (PD-1) and PDCD1LG2 (PD-L2), and a lower mean level of T cell signature based on expression of 13 T cell transcripts. (**b**) Control and NSD1 shRNA knockdown HNSC cells (1 × 10^6^) were injected into the subcutaneous compartments of the flanks of NOD-scid IL2Rgamma^null^ (NSG) mice. In each mouse, one flank was injected with control cells (black) and the other with NSD1 knockdown cells (red). After tumors were established (5 mm diameter), 100 × 10^6^ Ficoll-purified human PBMCs per mouse were injected via tail vein. After 10 days, tumors were dissociated, and tumor-infiltrating T cells (CD45^+^CD3^+^) were quantified by FACS. Cohorts were n = 5 per set of control and knock-down cell line, as indicated. *P < 0.05; **P < 0.005 (paired two-tailed Students t-test, error bars represent S.D.). (**c**) *NSD1 knockdown in HNSC results in the decreased expression of multiple chemokine genes*. Control and NSD1 shRNA knockdown HNSC cells were assessed for the expression of chemokine and chemokine-related genes using a qRT-PCR array. Log2 fold expression of of 35 chemokine-related genes upon NSD1 knockdown (Relative expression NSD1-shRNA/Control) in three established HNSC cell lines (PCI13, FADU, SCC6). Log2 fold expression is indicated by a color gradient, with NA values indicated in grey. Asterisks indicate genes that were upregulated* or downregulated** in the NSD1 subtype (relative to other subtypes) in the TCGA study.

We recently reported that levels of tumor associated leukocytes (TALs), inferred from gene expression data using the CIBERSORT algorithm^35,36^, varied between HNSC DNA methylation subtypes^22^ (Fig. 3a). The NSD1 subtype displayed an ‘immune cold’ subtype, displaying the lowest overall TAL levels as well as the lowest levels of specific TAL types including pro-inflammatory M1 macrophages, CD8^+^ cytotoxic T cells and resting CD4^+^ memory T cells, while plasma cells were highest within the NSD1 subtype.

Interestingly, the NSD1 subtype displayed low RNA expression of genes of relevance to immunotherapy, including the immune checkpoint receptor *PDCD1* (encoding PD-1), as well as its ligands *CD274* (encoding PD-L1) and *PDCD1LG2* (encoding PD-L2) (Fig. 3a).

It is widely understood that PD-1 expressed on CD8^+^ T cells binds PD-L1 and/or PD-L2 expressed on tumor cells or other cells within the microenvironment, resulting in suppression of anti-tumor immune response. A recent report indicates that PD-1 is also expressed on tumor associated macrophages (TAMs), that the PD-1/PDL1 checkpoint inhibits phagocytosis of tumor cells by TAMs, and that PD-1-PDL1 blockade immunotherapy functions through reactivation of TAMs as well as CD8^+^ T cells^37^. The authors reported that PD-1 is particularly expressed on alternatively activated M2, rather than classically activated M1 TAMs, based on cell surface protein markers. The co-occurrence of low *PDCD1* expression and M1, but not M2 TAM levels in the NSD1 subtype led us to hypothesize that PD-1 expression may actually be associated with M1 TAM levels; therefore, we investigated the correlation of PD-1 expression with different TAM fractions inferred by CIBERSORT, across 28 TCGA cancer types. Indeed, *PDCD1* expression was positively correlated with M1 macrophage and CD8^+^ T cells (Supplementary Figure 6). This postulates that M1 TAMs represent the TAM fraction that express PD-1 and are susceptible to reactivation by immunotherapy. Consistent with recent reports that TAMs are reprogrammed to express PD-L1^38–40^, M1 macrophage levels were also specifically correlated with expression of *CD274* and *PDCD1LG2* (Supplementary Figure 6). Given that both M1 TAMs and CD8^+^ T cells, as well as that *PDCD1*, *CD274* and, *PDCD1LG2* are lowest within the NSD1 HNSC subtype, we speculate that the NSD1 subtype is particularly immune evasive, and may be highly resistant to immunotherapy.

Using NSD1 RNA expression as a measure of NSD1 proficiency, we next validated the correlation of NSD1 expression with tumor infiltrating T cell levels in three independent primary HNSC population data sets, including the aforementioned GSE33232 data set and two additional datasets: GSE65858 (n = 253)^41^ and GSE39366 (n = 138)^26^. As a marker of T cell infiltration, we used a T cell signature based on mean expression of 13 T cell transcripts, previously employed elsewhere^42^. NSD1 RNA expression was positively correlated with T cell levels in all three independent patient cohorts, although the correlation was not statistically significance in the smallest (GSE33232) data set (Supplementary Figure 7). This indicates that NSD1 expression represents a reproducible marker of T cell infiltration in HNSC.

### Knockdown of NSD1 in HNSC results in immune cell exclusion from the tumor microenvironment

To assess a potential functional role of NSD1 inactivation in the exclusion of immune cells from the tumor microenvironment, we inhibited the expression of NSD1 by shRNA transduction in three established HNSC cell lines, PCI-13, FaDu, and UM-SCC-6. Matched sets of control and NSD1 knockdown cells were used to establish tumors in opposite flanks of immunodeficient NOD-scid IL2Rgamma^null^ (NSG) mice (Fig. 3b). Once tumors formed, human peripheral blood mononuclear cells (PBMCs) were injected intravenously, and the degree of T cell infiltration into the tumors was assessed by dissociation of the tumors and analysis of infiltrating T cell levels by flow cytometry. There was a significantly lower number of T cells in the NSD1 knockdown tumors compared to the control transduced tumors established from the three sets of cell lines. This points to a functional role of NSD1 inactivation in the exclusion of immune cells from the tumor microenvironment and is consistent with our observations of a correlation between NSD1 expression and T cell infiltration (Fig. 3a and Supplementary Figure 7)To begin to understand how NSD1 inactivation may be affecting T cell infiltration, we compared the expression of an array of chemokine genes in the control cell lines to matched NSD1 knockdown cell lines. The expression of multiple key chemokines important for immune cell recruitment was downregulated in the NSD1 knockdown cells (Fig. 3c), consistent with the reduction in the number of infiltrating T cells in NSD1 knockdown tumors. Thus, our data support a role of NSD1 as a tumor cell-intrinsic determinant of T cell infiltration into the tumor microenvironment.

## Discussion

Here we have described a hypomethylated, immune cold subtype of HNSC that is enriched for NSD1 mutations and somatic deletions, as well as a molecularly similar subtype in LUSC.

Our analysis indicates that both NSD1 mutations and deletions contribute significantly and independently to genome-wide deregulation of DNA methylation and transcription in a significant proportion of HNSCs and LUSCs. Indeed, our findings suggest that the most pronounced hypomethylation occurs due to biallelic loss of NSD1 at the transcriptional level, associated with combined mutations and deletions. Detailed genetic studies will be required to definitively characterize pathogenic lesions.

The NSD1 subtypes of HNSC and LUSC are characterized by DNA hypomethylation of many genes, concurrent with hypermethylation of a smaller set, resulting a net loss of ‘global’ DNA methylation. This indicates that NSD1 inactivation does not simply preclude DNA methylation, but alters its distribution, and implies a complex role of NSD1 in locus-specific epigenetic regulation.

An emerging consequence of cancer DNA hypomethylation is loss of epigenetic repression of developmental or germline tissue-specific genes, pushing cells to a more stem-like transcriptional profile^43,44^. This is apparent in NSD1-inactivated squamous cell carcinoma subtypes, where concurrent hypomethylation and overexpression of developmental transcription factors such as *PIWIL2* ^43^, *ELF5* ^32^, *TBX6* ^33^, and *FOXH1* ^34^ occurs. Such ectopically expressed genes may play oncogenic roles, as *PIWIL2* and *ELF5* represent epigenetically-regulated oncogenes that promote oncogenic transcriptional networks in lung and other cancers^30,31,45–47^. *PIWIL2* is among 31 genes that were hypomethylated in HNSC, LUSC, and Sotos syndrome, raising the intriguing possibility that genes and pathways that are responsible for overgrowth and cancer susceptibility in Sotos syndrome also promote growth in sporadic cancers.

NSD1 inactivation likely deregulates DNA methylation indirectly through alteration of underlying chromatin marks, as is the case of mutations in *SETD2* and MLL enzymes^48,49^. NSD1 inactivation could deregulate DNA methylation by impairing H3K36 trimethylation (H3K36me3), a mark that regulates DNA methylation^50–52^, as H3K36me1 and H3K36me2, the presumed methyltransferase products of NSD1^17–19^, represent substrates for conversion to H3K36me3 by SETD2^53,54^. Consistently, some^10,11,53^, though not all^19^ studies have found that NSD1 inactivation results in H3K36me3 loss. Interestingly, *SETD2* mutations, resulting in redistribution of H3K36me3, cause DNA hypermethylation at gene bodies in renal cell carcinoma^51^, contrasting with widespread promoter hypomethylation in NSD1-inactivated cancers.

It is generally understood that HNSC and LUSC are molecular similar, as these cancer types tend to cluster together in pan-cancer unsupervised clustering analyses^21,55,56^. Our analysis revealed a particularly striking correlation of the NSD1 subtypes between these two tumor types, postulating NSD1 inactivation as a driver of this novel molecular pan-cancer group. The defining feature of the NSD1 subtypes is likely to be loss of H3K36 methylation, resulting in altered DNA methylation and transcription. NSD1 genetic lesions represent one mechanism underlying impaired H3K36me; however, other mechanisms, such as H3K36 M mutations^19^ or those that impair NSD1 at the protein level, may account for H3K36me loss within the NSD1 wild type cancers within these subtypes.

Inference of TAL levels based on gene expression data revealed that the HNSC NSD1 subtype displays an ‘immune cold’ phenotype characterized by lower levels of overall TALs, and M1 TAMs, CD8+ T cells and resting CD4 memory T cells in particular. The correlation of *NSD1* RNA expression with a T cell signature was consistent in three independent patient cohorts.

Lower T cell levels within the NSD1 subtype are particularly clinically interesting, as T cell levels (particularly CD8^+^ T cells) represent markers of anti-cancer immune response that are associated with favorable prognosis in HNSC and other solid cancers^42,57–62^. Thus, our findings may have important implications for the future selection of immune therapy-responsive patients.

There is a growing interest in identifying the determinants of tumor immune infiltration, particularly of immune cell fractions that mediate anti-tumor immunity, such as CD8+ T cells and macrophages. Tumors can repress anti-tumor immune response by exploiting mechanisms of immune regulation, that normally function to prevent autoimmunity, such as by expressing ligands that activate immune checkpoints or by modulating expression of immune cells within the tumor microenvironment.

We have found intriguing evidence that NSD1 inactivation promotes immune evasion by the exclusion of immune cell infiltration into the tumor microenvironment. Using an *in vivo* model, we observed that the knockdown of NSD1 expression in HNSC tumors established in mice confers a decreased infiltration of CD8^+^ T cells compared to control tumors established in the same animals. The ability of a tumor cell-intrinsic driver to modulate the infiltration of immune cells into the tumor microenvironment has been demonstrated in melanoma, where β-catenin signaling has been shown to result in T cell exclusion, apparently through downregulation of the T cell attractant chemokine CCL4^42^. Moreover, PRC2 mediated epigenetic silencing or chemokines, associated with concordant promoter H3K27me3 and DNA hypermethylation, precludes T cell infiltration in ovarian cancer^63^. There was a significant reduction in the expression of several key chemokines associated with knocking down NSD1 in HNSC cell lines, indicating that NSD1 contributes to the regulated expression of these genes in the tumor cells. Efforts are underway to elucidate these mechanisms.

HNSC prognosis has shown little improvement in recent decades^4^. Immunotherapies such as monoclonal antibodies to PD-1 or PD-L1, which block the PD-1/PD-L1 checkpoint to restore anti-tumor immune response, are beneficial in a subset of HNSC cases, including metastatic or refractory HNSC cases^64^. There is a need to identify biomarkers to predict immunotherapy response, particularly as these treatments can cause autoimmune side effects^65^.

As the NSD1 subtype is depleted for both CD8^+^ T cells and PD-1 expressing TAMs, the HNSC NSD1 subtype may be particularly resistant to PD-1/PD-L1 checkpoint blockade immunotherapy, especially as immunotherapy response appears to be dependent on the presence of a preexisting immune cell population^66^. The mechanism by which NSD1 inactivation mediates immunosuppression remains to be determined. Most likely, NSD1 inactivation causes epigenetic deregulation of regulators of immune infiltration. Many such genes are epigenetically deregulated in the NSD1 subtype, representing a list of candidate immune regulators that may be investigated in future studies. Such immune regulators may include potential drug targets to restore anti-tumor immunity in NSD1 inactivated HNSCs.

Overall, this study reveals that *NSD1* inactivation is associated with widespread impairment of epigenetic regulation in both HNSC and LUSC, resulting in loss of epigenetic repression of potential oncogenes. In HNSC, NSD1 inactivation decreases immune cell infiltration, perhaps due to epigenetic deregulation of chemokines. Because this study was largely limited to analysis of existing data, we may have missed lesions in NSD1 or other genes due to data limitations, for example, we could not investigate the potential role of noncoding NSD1 mutations, as mutation data was generated using whole exome sequencing. Moreover, we did not have data for measures that could provide a more direct readout of NSD1 activity, such as NSD1 protein expression and histone methylation. Importantly, our findings are largely correlative; functional studies will be required to confirm causal roles of NSD1 inactivation in DNA hypomethylation immune evasion. Identification of the methyltransferase activity of NSD1 and classification of the pathways deregulated due to NSD1 inactivation may yield insight that could be exploited to develop novel targeted therapies.

## Methods and Materials

### Data processing

Preprocessed TCGA DNA methylation data (generated using the Illumina Infinium HumanMethylation450 and the HumanMethylation27 BeadChip arrays), gene expression data (generated by RNA sequencing), DNA copy number data (generated by microarray technology), and somatic point mutation data (generated by genome sequencing) were downloaded using the Firehose pipeline (version 2014071500 for gene expression and version 2014041600 for all other data sets)^67^. Copy number was called using GISTIC2.0. RNA-Seq data was processed using RSEM. Preprocessing for these data sets was done according to the Firehose pipelines described elsewhere^67^. Mutation data was accessed as Mutation Analysis reports, generated using MutSig CV v2.0^68^. Mutations predicted as silent by MutSig CV were removed. Additional data preprocessing of gene expression and DNA methylation data was done as follows: Genes and patients with more than 10% missing values for gene expression, and more than 20% missing values for DNA methylation, were removed. All remaining missing values were estimated using KNN impute^69^. Batch correction was done using Combat^70^.

### Classification of abnormally methylated genes

To reduce multiple testing of highly correlated CpG probes, probes for each gene were clustered using hierarchical clustering with complete linkage, and mean methylation (beta-value) was calculated for each CpG cluster. MethylMix (Version 1.6.0) was applied to CpG cluster data to systematically identify regional CpG clusters that are abnormally methylated in cancer versus normal tissue, where DNA methylation is inversely associated with RNA expression of the corresponding gene, using beta-mixture models, as previously described^71^. For each gene (CpG cluster), MethylMix ascribes either normal or abnormal (hypomethylated or hypermethylated) DNA methylation states to each patient. For LUSC, 370 patients had DNA methylation data generated using the Illumina 450 k array, while 133 patients had methylation data measured using the Illumina 27 k array. To maximize the methylation data in terms of either patient numbers or genomic coverage, depending on the application, MethylMix was applied twice: first to all 503 patients, using data for CpG probes that were shared between the 450 k and 27 k array platforms (n = 23,362 probes), and then to separately the 370 patients with 450 k array data (n = 395,772). For HNSC, all 528 patients had DNA methylation data generated using the Illumina 450 k array.

### Consensus clustering of abnormally methylated genes

Consensus clustering was applied to MethylMix output data, i.e. methylation state data, for cancer patients, to identify robust patient clusters (Putative subtypes). Consensus clustering was performed using the ConsensusClusterPlus R package^72^ (Version 1.36.0), with 1000 rounds of k-means clustering and a maximum of k = 10 clusters. Selection of the best number of clusters was based on visual inspection ConsensusClusterPlus output plots. For HNSC, subtypes are as previously described^22^. For LUSC, consensus clustering was applied to MethylMix output data for all 503 patients, in order to maximize the number of patients with both mutation and DNA methylation data.

### Identification of genes associated with NSD1 subtypes

SAM analysis^73^ was used to identify genes that were overexpressed and underexpressed NSD1 subtypes relative to all other patients (Using the samr package for R (Version 2.0)). SAM analysis was also used to identify genes (CpG clusters) that were either hypermethylated or hypomethylated within the NSD1 subtypes, using mean methylation for each CpG cluster. For LUSC, SAM analysis was applied only to DNA methylation data for he 370 patients with 450 k array data (Excluding patients wit 27 k data), to maximize genome coverage.

### Centroid-based classification of LUSC patients to the HNSC NSD1 subtype

Prediction Analysis of Microarrays (PAM)^27^ was used to develop a DNA methylation classifier to predict the HNSC NSD1 subtype, and to classify LUSC patients that are most similar to the HNSC NSD1 subtype at the level of DNA methylation. Briefly, PAM uses a nearest shrunken centroids method to assign the class of each LUSC patients based on the squared distance of the DNA methylation profile for that individual to the centroids of known class groups (i.e. HNSC patients within, or not within the NSD1 subtype).

We applied PAM to DNA methylation data for all 10,818 CpG sites within all gene regions that were abnormally methylated (Hypomethylated or hypermethylated) in HNSC, identified using MethylMix^71^, as previously reported^22^. PAM analysis uses Shrinkage to select the optimum number of CpG probes for class prediction, such that the model selects only a subset of CpG probes to develop the centroids. We first used PAM in combination with 10-fold cross validation to determine the ability of the DNA methylation data to predict the NSD1 subtype within TCGA data. For each fold of cross validation, the PAM model was trained on 90% of patients and assigned class probability for belonging to the NSD1 subtype to the each of the remaining 10% of patients based on the distance of the patient to its closest centroid. We used the Area under the ROC curve (AUC) to evaluate the performance of the model in accurately predicting the class of samples. We then applied this DNA methylation classifier signature to 365 TCGA LUSC patients (All patients with 450 k array data) to classify them into either a ‘HNSC NSD1 subtype’ class or the ‘HNSC other subtype’ class. We only used classification results when probabilities were >60% or <40%, excluding low confidence assignments for one borderline individual from analyses. PAM analysis was performed using the pamr R package (Version 1.55).

### Testing overlap between the DNA hypomethylation signatures associated with NSD1 inactivation in cancer and Sotos syndrome

The Sotos syndrome overlap index was calculated for each patient as follows: For each patient, we generated a list of all hypomethylated CpG probes, i.e. the CpG probes within all hypomethylated genes (Genes with a hypomethylated tumor state, identified by MethylMix). For a given patient, the Sotos syndrome overlap index represent the number of these hypomethylated CpG probes that overlaps/intersect with the Sotos syndrome hypomethylated CpG signature, divided by the number of all hypomethylated CpG probes.

The random overlap index was generated for each patient using the same calculation, except replacing the Sotos syndrome hypomethylated CpG signature with a random signature, i.e., a set of randomly selected CpGs (using the ‘sample’ base function within R) of the same length as the Sotos syndrome hypomethylated CpG signature. To control for sampling error, we calculated the random overlap index ten times, each time generating a new random signature, and the mean of these ten iterations as the final random overlap index.

The NSD1 lesion score calculated by adding the number NSD1 mutations to the additive inverse of the Gistic2.0 copy number score. This represents an approximation of the number of inactivating NSD1 lesions, where scores of 1 or 2 may correspond to inactivation of one or both NSD1 alleles, respectively. This represents an approximation, particularly because the Gistic2.0 score represents an approximation of NSD1 copy number.

### Inference of tumor associated leukocyte levels

CIBERSORT (Version 1.03) was applied to gene expression (RNA-Seq) data to infer the levels of specific TAL types, as previously described^35,36^. Only patients for with estimation p-values less than 0.05 (n = 309 of 520 patients with RNA expression data), indicating high confidence TAL estimation, were included in downstream analyses.

### Inference of infiltrating T cells using a T cell gene expression signature

Mean expression of a set of 13 T cell transcripts (*CD8A*, *CCL2*, *CCL3*, *CCL4*, *CXCL9*, *CXCL10*, *ICOS*, *GZMK*, *IRF1*, *HLA-DMA*, *HLA-DMB*, *HLA-DOA*, *HLA-DOB*), across all 13 genes, was used as a method of inferring relative T cell levels, as previously described^42^. This T cell score was strongly correlated with expression of CD8+ T cell expressed *PDCD1* and negatively associated with expression of *EPCAM*, a marker of epithelial tumor purity (Low stromal/immune content)^74^ (Supplementary Figure 8).

### Processing copy number data (For the GSE33232 study cohort)

Raw CEL signal intensity files (Generated using the Affymetrix Genome-Wide Human SNP 6.0 Array) were processed with Affymetrix power tools and BIRDSUITE (Version 1.5.5)^28,75^. Segmented copy-number calls were log2 transformed and further processed with GISTIC 2.0^24^ using an amplification and deletion threshold of 0.1. Samples with *NSD1* copy number calls meeting the GISTIC 2.0 (Version 2.0.0) threshold and designated at least −1 or +1 were considered to have *NSD1* deletions and amplifications, respectively.

### Mice and cell lines

NSG mice (NOD-scid IL2Rgamma^null^, 6–12 weeks old) on a C57BL/6 background were a kind gift from Dr. Ravi Majeti (Stanford University) and were bred in our animal facility under pathogen-free conditions. The protocols used in this study were approved by the Administrative Panel on Laboratory Animal Care (APLAC) at Stanford University (Stanford, CA). All methods were performed in accordance with this protocol, and with the ALPAC guidelines and regulations.

The human HNSC cell lines PCI-13 was a gift of Suzanne Gollin at the University of Pittsburgh. The UM-SCC-6 cell line was obtained from the University of Michigan. The FaDu cell line was obtained from ATCC. Cells were maintained in complete DMEM:F12 medium (DMEM:F12 1:1 with 10% heat- inactivated FBS [Omega Scientific], 100 IU/ml penicillin and 100 μg/ml streptomycin [Gibco, Invitrogen, CA]). The 293 T cell line was obtained from ATCC and maintained in complete DMEM medium. Culture medium was changed every 2–3 days depending on cell density, and subculture was conducted when confluence was reached.

### Lentiviral shRNA transduction

For the production of the lentiviral particles, 293 T cells were transfected using Lipofectamin2000 (Invitrogen) with the packaging plasmid pCMVR8.74 (Addgene), the envelope plasmid pCMV-VSVG, and the lentiviral construct containing the human NSD1 shRNA (pGIPz lentiviral vector, Dharmacon GE Life Sciences). Cell culture medium was changed 16 hours after the transfection and virus supernatants were collected 24 and 48 hours after the media change. Immediately after supernatant collection, the viral particles were concentrated by polyethylene glycol precipitation with PEGit solution (SBI Bioscience), according to the manufacturer’s protocol. The lentiviral pellets were then resuspended in ice-cold PBS. For the lentiviral transduction of the cell lines, cells were plated at the appropriate concentration (1 × 10^5^ cells per 6 well plates). Then, the lentiviral particles were added to the cell cultures at a multiplicity of infection (MOI) of 1 transducing Unit per cell. Polybrene (8 ug/ml) was also added to enhance the lentiviral transduction efficiency. Medium was changed 24 hours after viral infection. All transfected cells were purified by FACS sorting for GFP^+^ cells and expanded for the experiments.

### RNA extraction and chemokine gene expression array

RNA was extracted with the RNeasy mini kit (QIAGEN), and cDNA made with the Maxima First Strand cDNA Kit (ThermoFisher Scientific). For chemokine gene expression assessment, a TaqMan human chemokine and cytokine array was purchased from ThermoFisher Scientific and was used per the manufacturers protocol. The amplified cDNA was diluted with nuclease-free water and added to the TaqMan® Gene Expression Master Mix (ThermoFisher Scienticfic). Then, 20 μl of the experimental cocktail was added to each well of the TaqMan™ Array Human Chemokines (ThermoFisher Scienticfic, CA). Real-Time PCR was performed on the 7900HT Fast Real-Time PCR System (Applied Biosystems, CA) with the following thermal profile: segment 1–1 cycle: 95 °C for 10 minutes, segment 2–40 cycles: 95 °C for 15 seconds followed by 60 °C for 1 minute, segment 3 (dissociation curve) −95 °C for 1 minute, 55 °C 30 seconds, and 95 °C for 30 seconds. Expression of cytokines relative to the HPRT reference gene are shown in Supplementary Table 4. Genes that were not detected, or with extremely low expression values relative to HPRT (<0.001) were excluded from analysis.

### *In vivo* tumor infiltration assay and flow cytometry

Control and NSD1 shRNA knockdown HNSC cells (1 × 10^6^) were injected into the subcutaneous compartment of the flanks of NSG mice. In each mouse, one flank was injected with control cells and the other with an equal number of NSD1 knockdown cells. After tumors were established (5 mm diameter), 100 × 10^6^ Ficoll-purified human PBMCs per mouse were injected via tail vein. After 10 days, tumors were dissociated, and tumor-infiltrating T cells (CD45^+^CD3^+^) were quantified by FACS. De-identified human PBMCs were obtained from the Stanford Blood Center (Palo Alto, CA), in accordance with an Institutional Review Board (IRB)-approved protocol, and prepared by Ficoll gradient centrifugation (GE Healthcare, Piscataway, NJ, USA). For tumor digestion, tumors were isolated/minced and digested in 300 U/mL collagenase and 100 U/mL hyaluronidase (StemCell Technologies) in culture media; DMEM/F-12 medium (Corning) with 10% FBS, and 1% penicillin-streptomycin-amphotericin B (ThermoFisher Scienticfic). The tumor digest was pipetted every 15 minutes and incubated at 37 °C for 3 hours, until a single-cell suspension was obtained. The dissociated cells were spun down and resuspended in Trypsin/EDTA (StemCell Technologies) for 5 minutes, then further dissociated with 5 U/mL dispase (StemCell Technologies) and 0.1 mg/mL DNase I (StemCell Technologies) for 1 minute. Cells were filtered through a 40-mm cell strainer and erythrocytes were lysed with ACK lysing buffer (Lonza) prior to antibody staining and FACS. The dissociated cells were resuspended in ice cold FACS solution (PBS supplemented with 2% fetal calf serum and 1% penicillin-streptomycin) and stained with PerCP-Cy5.5-anti-human CD3, APC-anti-human CD45 (BioLegend, CA) according to the manufacturer’s protocols. DAPI (1 μg/mL) was added to all the tubes prior to filtering through a 70 μm membrane. Labeled cells were analyzed on a FACSAria III (BD Biosciences).

### Data Availability Statement

All data generated or analyzed during this study are included in this published article (and its supplementary information files).

### Source code

Code used for analyses associated with this report are available as R scripts at: https://github.com/kevinbrennan/NSD1_10032017/blob/master/Code_Github_NSD1_paper.R

## Electronic supplementary material

Supplementary Information

Supplementary Dataset 1

Supplementary Dataset 2

Supplementary Dataset 3

Supplementary Dataset 4
