# Supplementary material for: NSD1 inactivation defines an immune cold, DNA hypomethylated subtype in squamous cell carcinoma
Source: Sci Rep. 2017 Dec 6;7:17064. doi: 10.1038/s41598-017-17298-x (PMC5719078; doi:10.1038/s41598-017-17298-x)

NSD1 inactivation defines an immune cold, DNA hypomethylated subtype in squamous cell carcinoma

Kevin Brennan[1*], June Ho Shin[2*], Joshua K. Tay[2,4], Marcos Prunello[3], Andrew Gentles[1], John B. Sunwoo[2*^], Olivier Gevaert[1*^]

[*]These authors contributed equally

[*^]Co-corresponding authors

Supplementary figure legends

Supplementary figure 1: Association of NSD1 inactivating lesions with NSD1 RNA expression and genome-wide abnormal DNA methylation. Levels of a) *NSD1* expression (RNA-Seq) and b) genome-wide DNA methylation, within patient stratified by *NSD1* somatic copy number (Panels) and number of *NSD1* mutations (Colored boxes), indicated for head and neck squamous cell carcinoma (HNSC) and lung squamous cell carcinoma (LUSC) separately. Asterisks indicate statistical significance of difference in *NSD1* expression between indicated groups. 'Genome-wide abnormal DNA methylation' represents mean DNA methylation across all abnormally methylated genes, i.e. genes that were either hypermethylated or hypomethylated in tumor relative to normal tissue. *Wilcoxon rank sum test p-value <0.05. ***Wilcoxon rank sum test p-value <0.001.

Supplementary figure 2: Pan-cancer analysis of DNA hypomethylation associated with NSD1 mutations. Boxplots indicate the number of hypomethylated genes, stratified by *NSD1* mutation status (i.e. number of *NSD1* mutations), for three cancer types (TCGA cancers for which there were at least 10 patients with *NSD1* mutations and DNA methylation data). These include lung squamous cell carcinoma (LUSC, n=10 patients with *NSD1* mutations), uterine corpus endometrial carcinoma (UCEC, n=25 patients with *NSD1* mutations), breast carcinoma (BRCA, n=11 patients with *NSD1* mutations).

Supplementary figure 3: Association of NSD1 RNA expression and copy number with PAM model classes in independent patient cohort. Patients within a validation set of 44 primary

HNSCs (GSE33232) were split into two groups based on class prediction for belonging to the NSD1 subtype, using a PAM model that was trained on TCGA DNA methylation data.
The asterisk represents the Wilcoxon rank sum test p-value for a difference in mean *NSD1* expression (RNA-Seq) between patients of the predicted NSD1 subtype class (red) and the other class (grey). *P value <0.05 & >0.01. Correlation of *NSD1* expression with DNA methylation of CpG sites that were i) hypomethylated (Supplementary table 1) and ii) hypermethylated (Supplementary table 2) in the TCGA NSD1 subtype, in the GSE33232 cohort. Selected hypomethylated (n=37) and hypermethylated (n=34) CpGs represent those that were within the top 100 genes that were most hypomethylated and hypermethylated genes in the NSD1 subtype relative to other subtypes, in the TCGA cohort. These CpG sites represented those that overlapped between the platforms used to measure DNA methylation in the GSE33232 (Illumina 27k array) and TCGA (Illumina 450k array) cohorts. Patients predicted as belonging to the NSD1 subtype based on the PAM model are highlighted in red. Linear regression P-values and Pearson correlation coefficients (cor) are indicated. *NSD1* copy number calls are, inferred using GISTIC2.0., are indicated by point shapes.

Supplementary figure 4: Overlap between aberrant DNA hypomethylation signatures associated with cancer and Sotos syndrome. a) Boxplots illustrate levels of the Sotos syndrome overlap index between patients groups stratified by the number of NSD1 genetic lesions including NSD1 mutations (illustrated by colored boxes) and NSD1 copy number aberrations (Gistic2.0. copy number calls, with copy number groups split by panels). Overlaps are shown in squamous cell carcinomas of the head and neck (HNSC) and lung (LUSC), separately. The Sotos syndrome overlap index represents a measure of similarity between the DNA hypomethylation signatures

of each patient tumor, and that of Sotos syndrome overall, and is calculated as the fraction of hypomethylated CpG in each patient tumor that overlap with a previously published Sotos syndrome DNA hypomethylated CpG signature (1). b) Overlap with a 'random overlap index' (A random control for the Sotos syndrome overlap index) is indicated, generated by replacing the Sotos syndrome hypomethylated CpG signature with a random CpG signature of the same length. The random overlap index illustrated represents the mean across ten iteration of random CpG selection and overlap calculation, to control for sampling error (See methods for details).

Supplementary figure 5: Enrichment of the Sotos syndrome DNA hypomethylated CpG signature In NSD1 inactivated cancers: a) Boxplots illustrate levels of i) the Sotos syndrome overlap index and ii) the random overlap index in patients stratified by the number of inactivating NSD1 lesions (NSD1 mutations and copy number aberrations combined into a single score (See methods for details)), in head and neck squamous cell carcinoma (HNSC) and lung squamous cell carcinoma (LUSC) separately. Asterisks beneath horizontal arrows indicate the significance of linear relationship between the Sotos syndrome overlap index and the NSD1 lesion score.
b) Boxplots illustrate levels of i) the Sotos syndrome overlap index and ii) the random overlap index, in each DNA methylation subtype of HNSC and LUSC, with NSD1 subtypes highlighted indicated in red (other subtypes grey). Asterisks indicate the level of significance of differences in levels of the Sotos syndrome overlap index between the NSD1 subtype and each other DNA methylation subtype (Wilcoxon rank sum test).
The Sotos syndrome overlap index represents a measure of similarity between the DNA hypomethylation signatures of each patient tumor, and that of Sotos syndrome overall, and is calculated as the fraction of hypomethylated CpG in each patient tumor that overlap with a

previously published Sotos syndrome DNA hypomethylated CpG signature (1). The random overlap index' represents a random control for the Sotos syndrome overlap index; it is generated by replacing the Sotos syndrome hypomethylated CpG signature with a random CpG signature of the same length. This random overlap index represents the mean across ten iteration of random CpG selection and overlap calculation, to control for sampling error (See methods for details). NS not significant, * $p<0.05$, ** $p<0.01$, *** $p<0.001$.

Supplementary figure 6: Correlation of PDCD1 (PD-1) expression with inferred levels of tumor associated leukocyte levels in 28 TCGA cancers. Heatmap indicates coefficients for correlation (Pearson) of *PDCD1* RNA expression with levels of CD8+ T cells, M0, M1 and M2 tumor associated macrophages (TAMs), in 28 cancers, using data derived from the TCGA study.

Supplementary figure 7: Validation of association of NSD1 RNA expression with a T cell transcript signature in independent patient cohorts. Association of a13 gene T cell transcript signature within the TCGA cohort (Shown for reference), and within gene expression datasets for three independent primary HNSC cohorts, including GSE65858 (n=253) [41], GSE39366 (n=138) and GSE33232 (n=44) [28].

Supplementary figure 8: Positive correlations of a T cell signature with expression of PDCD1 expression and inverse correlation with expression of EPCAM (RNA-Seq).

Supplementary tables

Supplementary table 1: Genes overexpressed/hypomethylated in NSD1 subtypes of HNSC and LUSC

Supplementary table 2: Genes underexpressed/hypermethylated in NSD1 subtypes of HNSC and LUSC

Supplementary table 3: CpGs used to predict the NSD1 subtype in HNSC NSD1 PAM model

Supplementary table 4: Cytokine RNA expression, relative to HPRT, in three cell lines

Supplementary figure 1

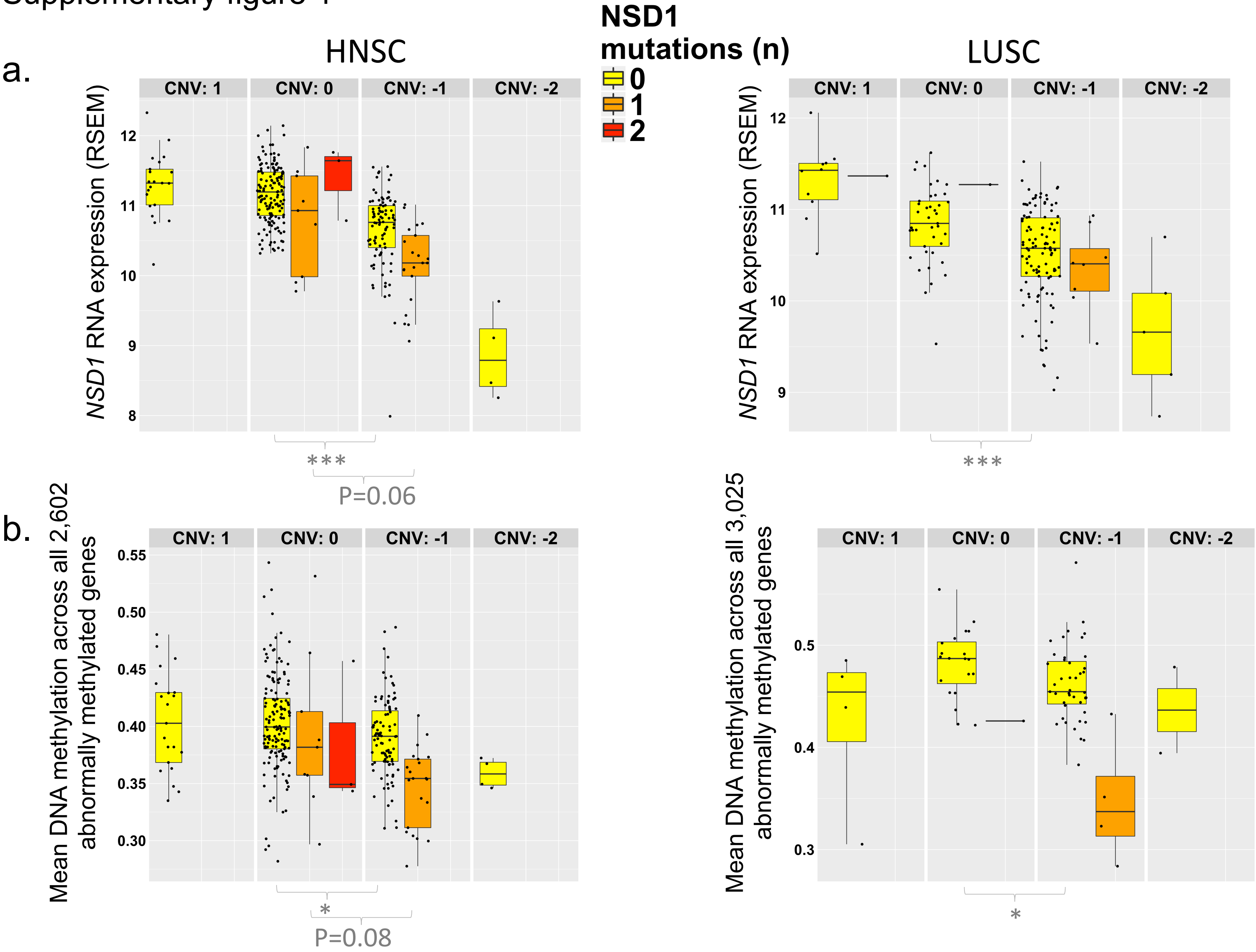

Supplementary figure 2

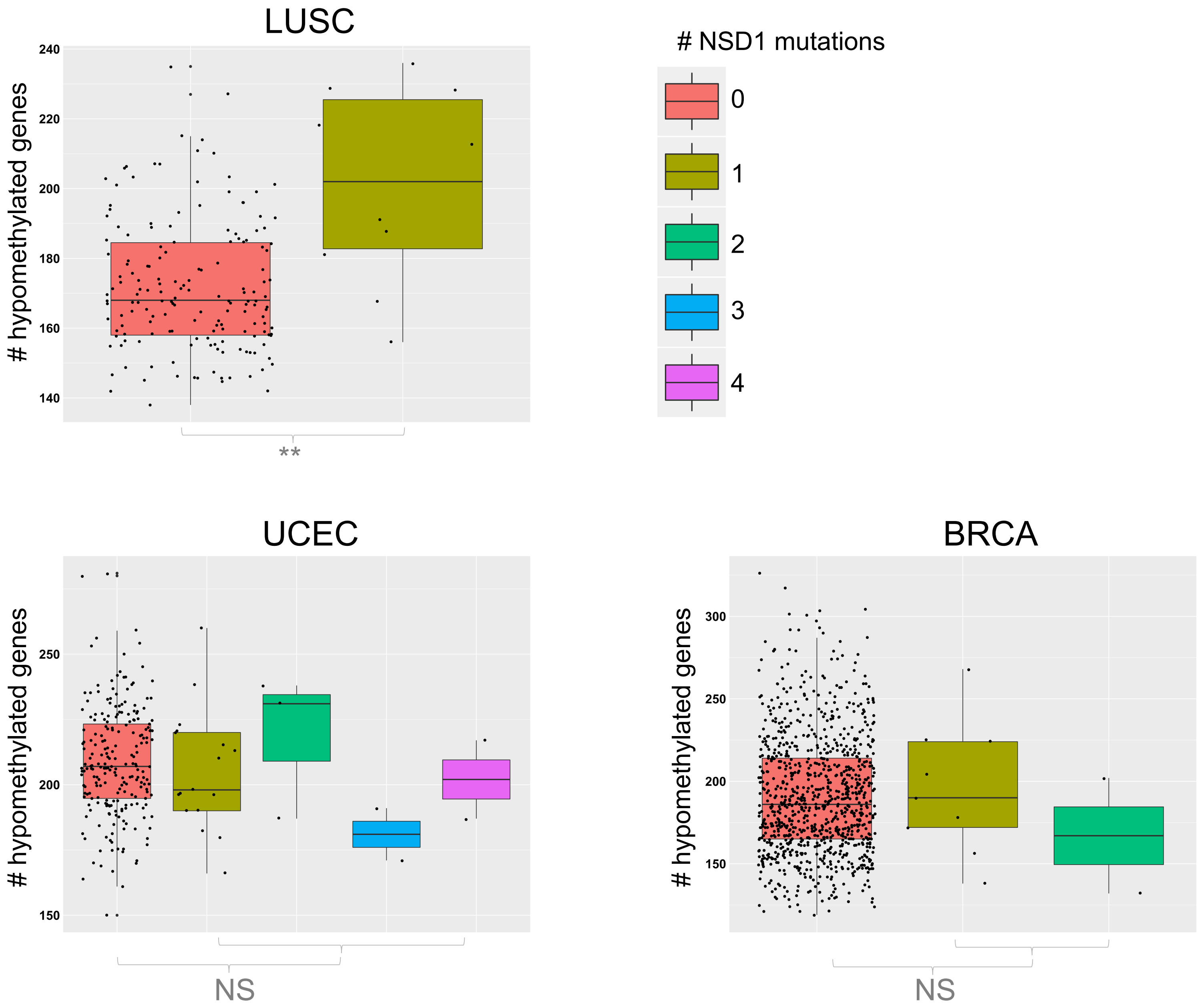

Supplementary figure 3

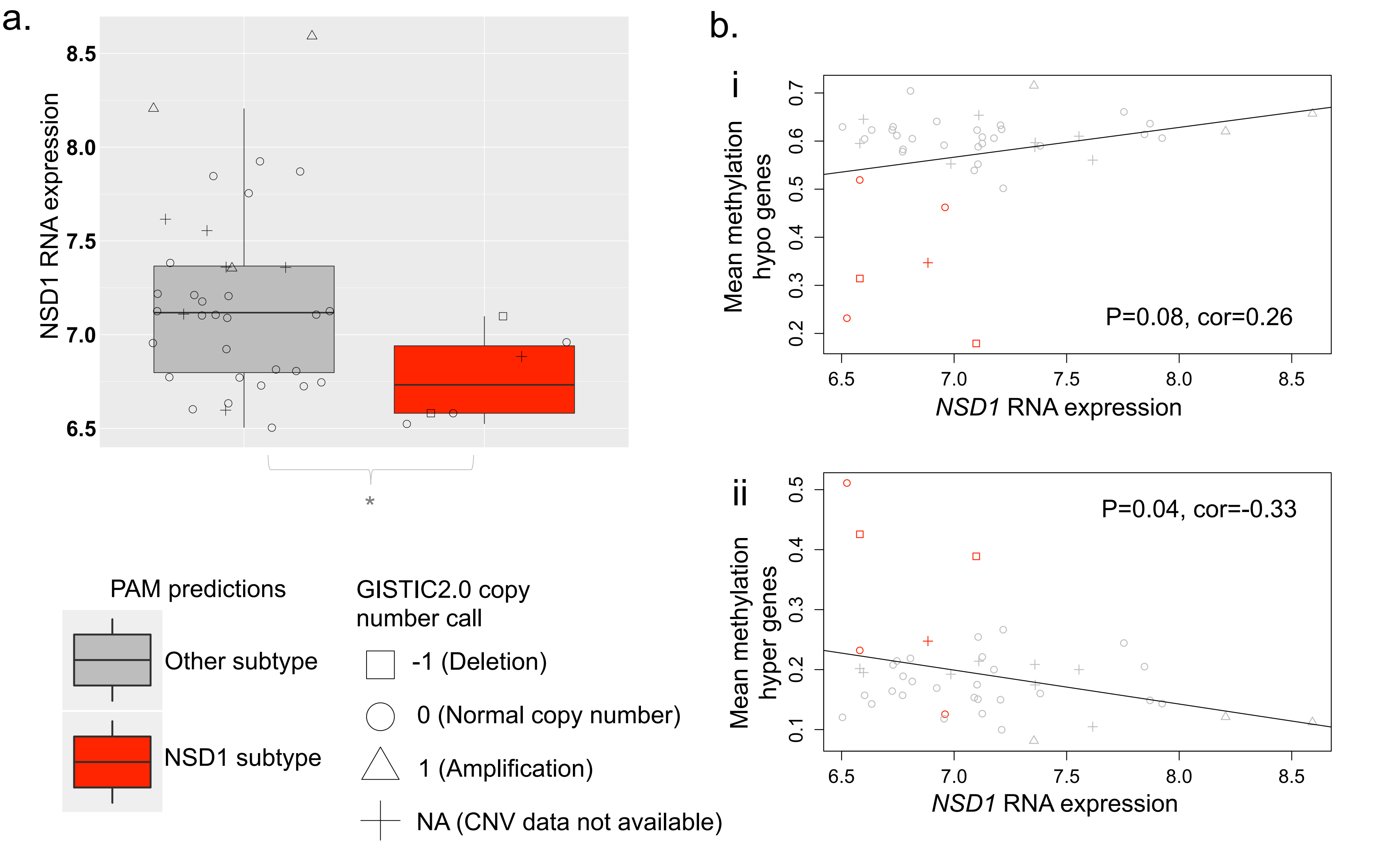

Supplementary figure 4

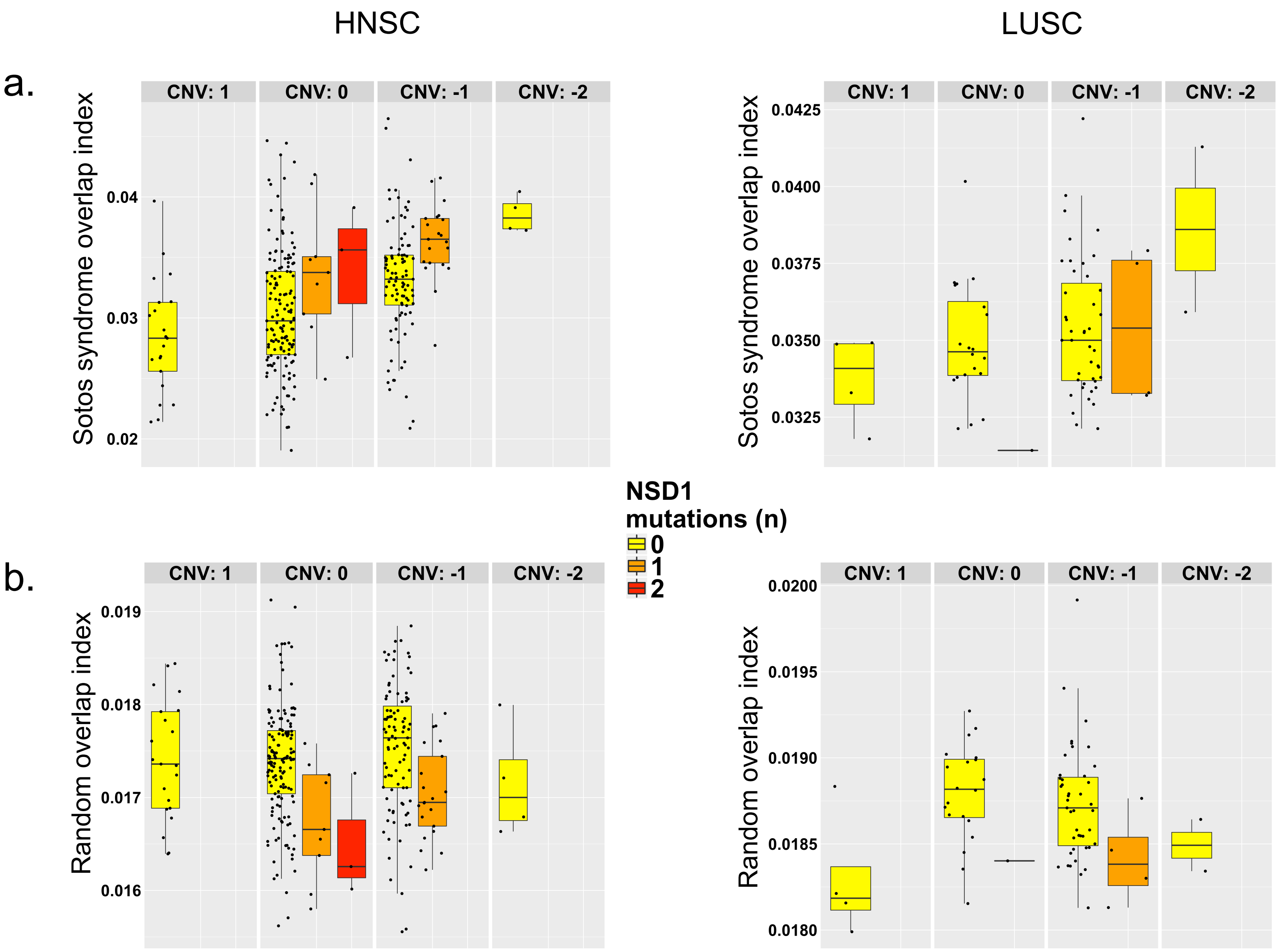

Supplementary figure 5

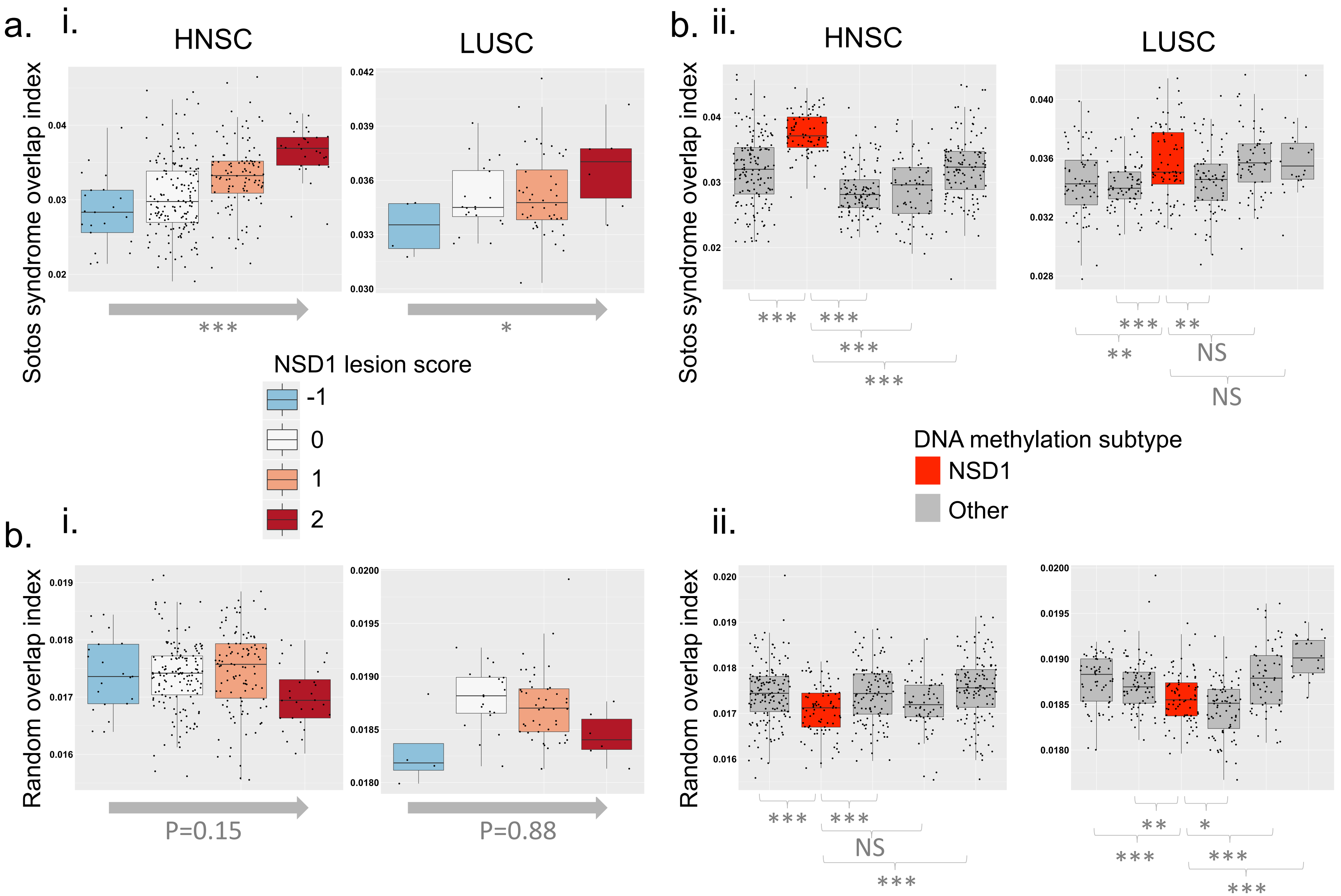

Supplementary figure 6

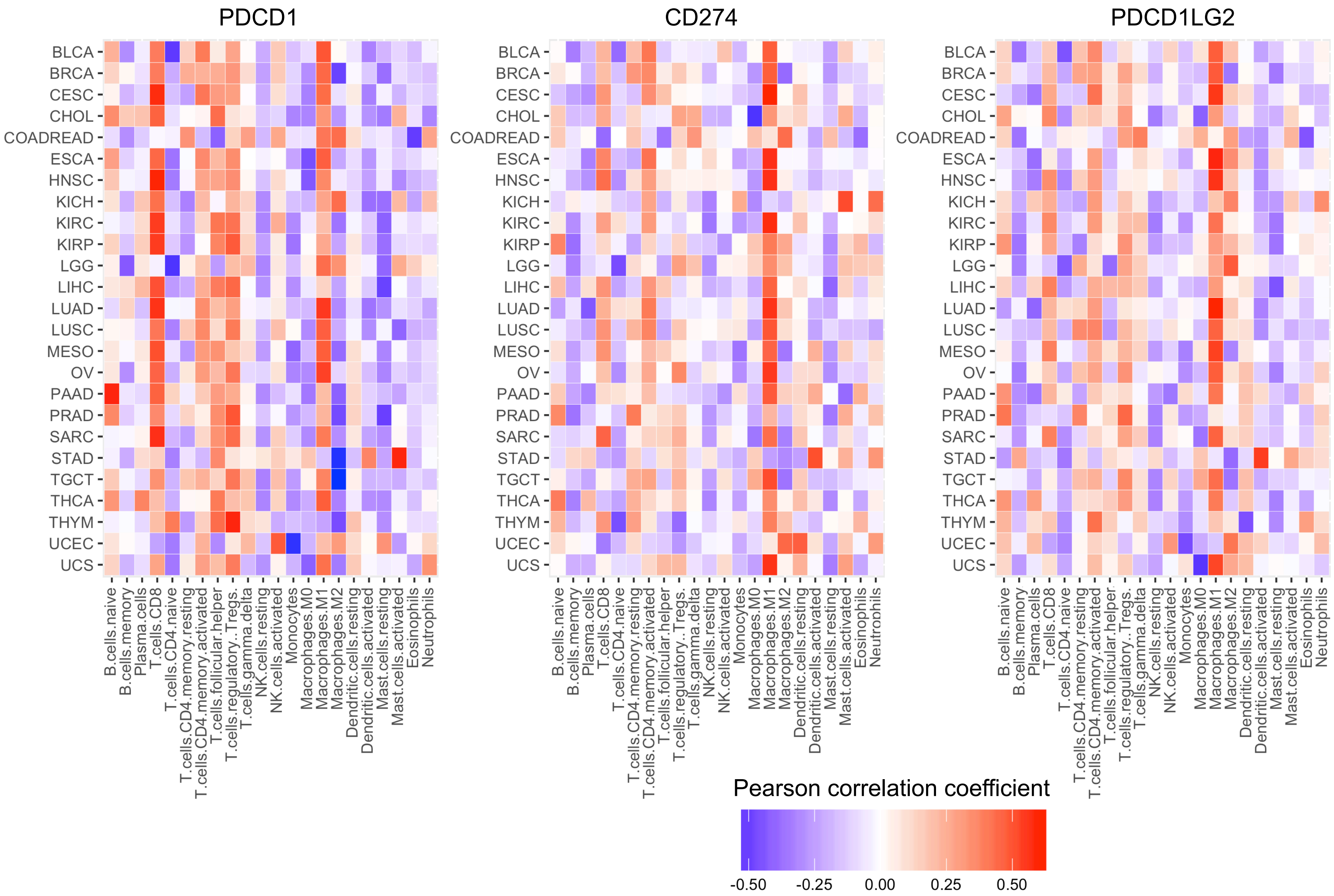

Supplementary figure 7

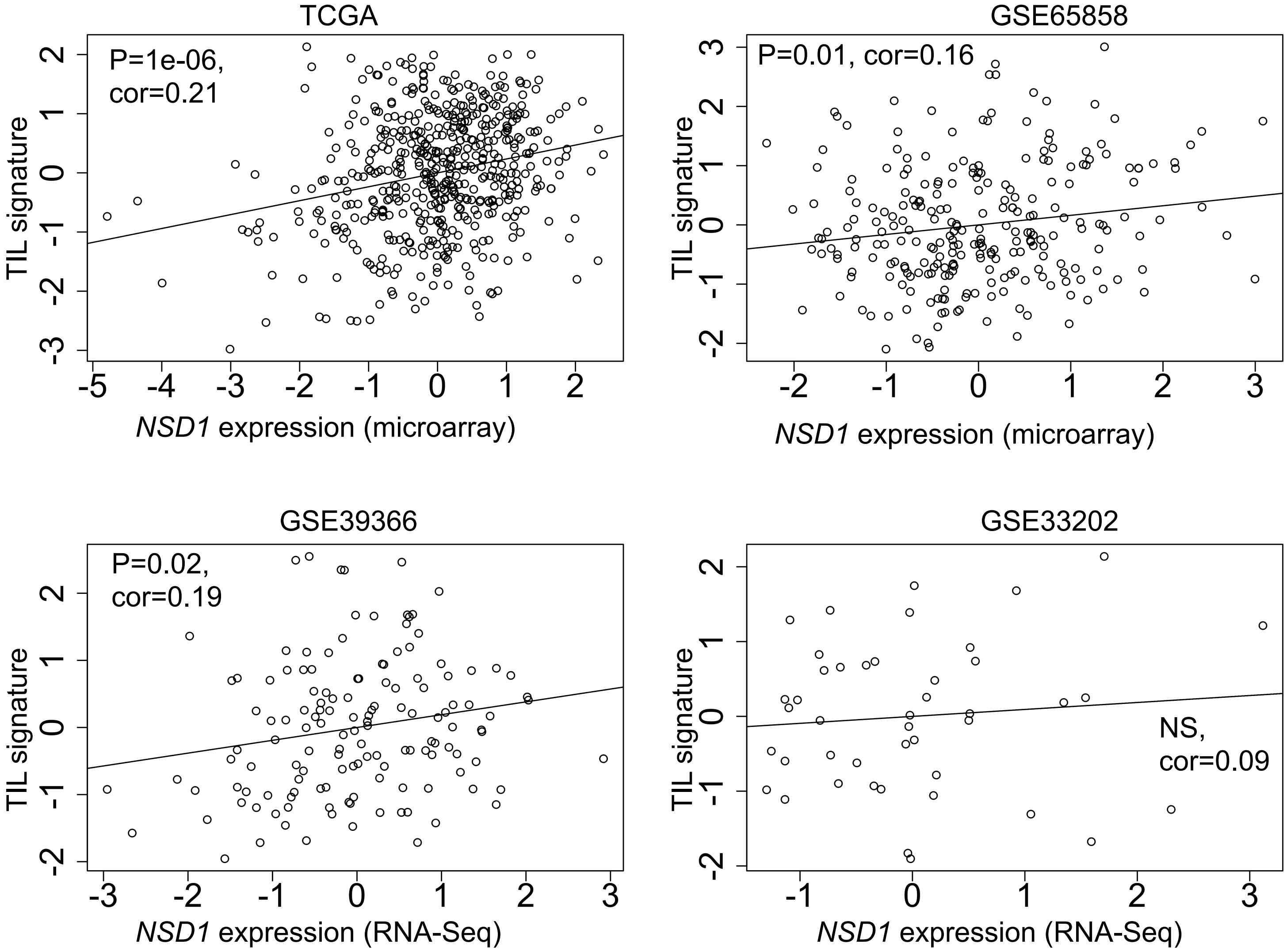

Supplementary figure 8

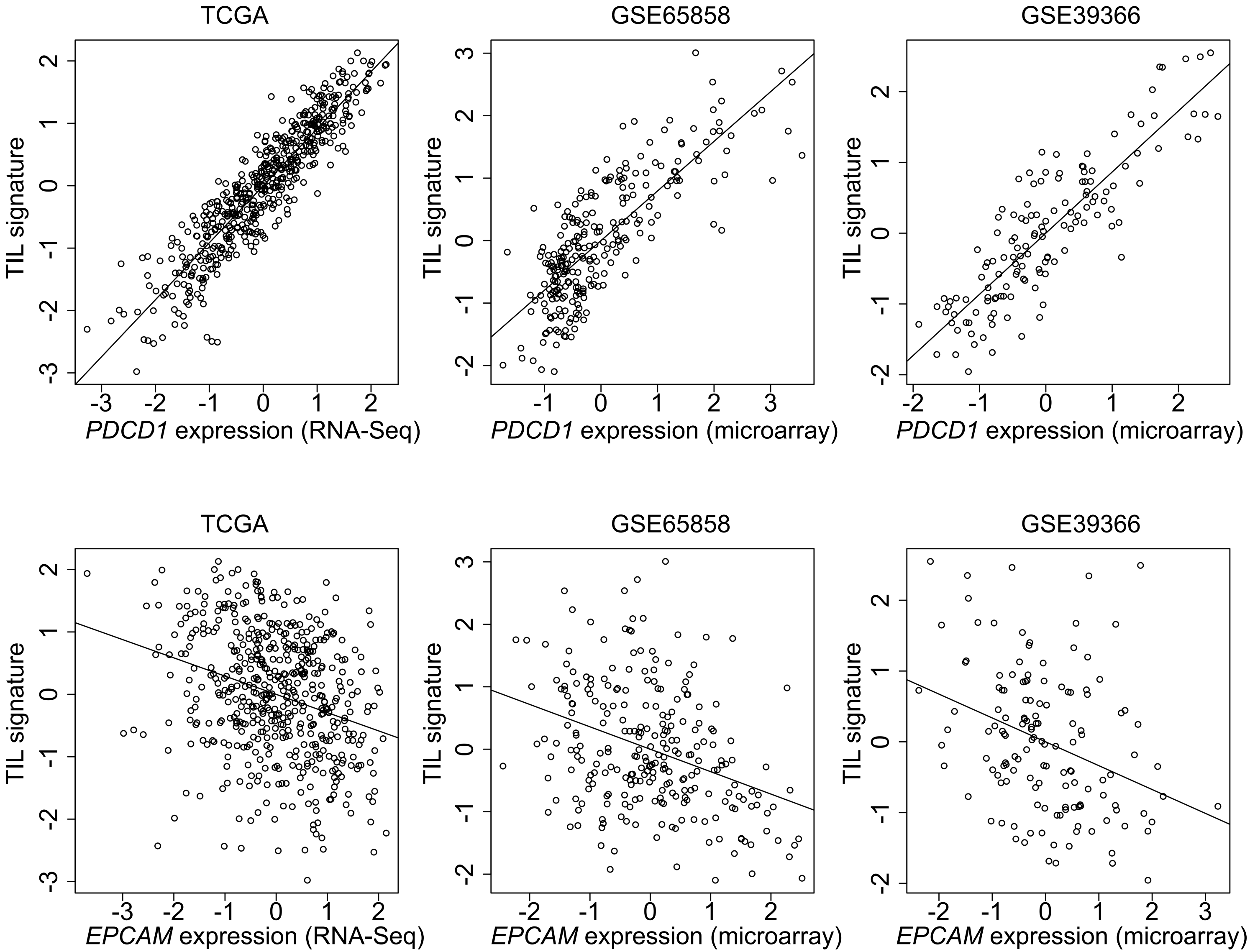

Supplement: Supplementary file 1 — Supplementary Information [file 41598_2017_17298_MOESM1_ESM.pdf]
